# Advances in Linking Wintering Migrant Birds to Their Breeding-Ground Origins Using Combined Analyses of Genetic and Stable Isotope Markers

**DOI:** 10.1371/journal.pone.0043627

**Published:** 2012-08-20

**Authors:** Amy A. Chabot, Keith A. Hobson, Steven L. Van Wilgenburg, Gregory J. McQuat, Stephen C. Lougheed

**Affiliations:** 1 Department of Biology, Queen’s University, Kingston, Ontario, Canada; 2 Environment Canada, Saskatoon, Saskatchewan, Canada; 3 Department of Geography, Queen’s University, Kingston, Ontario, Canada; Ohio State University, United States of America

## Abstract

An enduring problem in avian ecology and conservation is linking breeding and wintering grounds of migratory species. As migratory species and populations vary in the degree to which individuals from distinct breeding locales mix on stop-over sites and wintering grounds, establishing migratory connectivity informs our understanding of population demography and species management. We present a new Bayesian approach for inferring breeding grounds of wintering birds of unknown origins in North America. We incorporate prior information from analysis of genetic markers into geographic origin assignment based upon stable-hydrogen isotope analysis of feathers (δ^2^H_f_), using the Loggerhead Shrike (*Lanius ludovicianus*). Likely geographic origins derived from analyses of DNA microsatellites were used as priors for Bayesian analyses in which birds were assigned to a breeding-ground origin using their δ^2^H_f_ values. As with most applications of Bayesian methods, our approach greatly improved the results (i.e. decreased the size of the potential area of origin). Area of origin decreased by 3 to 5-fold on average, but ranged up to a 10-fold improvement. We recommend this approach in future studies of migratory connectivity and suggest that our methodology could be applied more broadly to the study of dispersal, sources of productivity of migratory populations, and a range of evolutionary phenomena.

## Introduction

Linking breeding and wintering grounds of populations, particularly for declining or endangered migratory animals, is a major endeavor for conservation agencies and scientists, yet one fraught with technical and logistical challenges. Migratory species and populations vary in the degree to which individuals from distinct breeding locales mix on different stop-over sites and wintering grounds. Therefore, to understand population demographics, local adaptation, and causes of diversification, the spatial characteristics (i.e. which populations mix on breeding and wintering grounds) and strength (i.e. proportion of individuals from distinct breeding areas on the wintering grounds, or *vice versa*) of migratory connectivity [1]–[3] must be quantified. In turn, this informs the effective, typically multinational, management strategies for migratory species [4] and provides insight into evolutionary trajectories [2].

Extrinsic markers (e.g. numbered leg bands) have provided the most direct empirical estimates of movements of migrants throughout the world [5], [6], but often require both capture and recapture of individuals, which limits the amount of data collected. Return rates are typically very low [5] and while this technique provides high spatial resolution, data are necessarily biased to origins where individuals are marked [7].

Intrinsic markers representative of the region of origin require only an initial capture, but to be of use, population-level differences in the marker must exist, and have been quantified *a priori*. For example, inherited phenotypic characteristics that show diagnostic geographic variation have also proven useful for distinguishing among breeding populations of some species on the wintering grounds [8], [9]; however not all species show distinct morphologies among populations or subspecies. While various intrinsic markers have been identified [7], the two most commonly used are genetic and stable isotope markers [10]–[13].

Analysis of stable-hydrogen isotopes in feathers is one of the most commonly applied intrinsic marker techniques in studies of migration [14]. The continent-wide pattern in mean growing-season stable-hydrogen isotope ratios (δ^2^H) in rainfall show strong and predictable spatial gradients. Hydrogen in precipitation is integrated into the tissues of an organism, and so consumer tissues ultimately reflect the H isotope composition of the hydrosphere driving the local foodweb. A number of assumptions are inherent in the application of H isotope measurements for the study of geographic connectivity [15]; principally that i. the annual variance in the deuterium isoscape is small compared to the magnitude of the isotopic gradient, ii. the algorithm linking avian tissues to the H precipitation isoscape are accurately estimated, and iii. sources of variance can be estimated and reflected in the assignment of individuals to their origins.

Genetic markers are also commonly used to infer origins of migratory species. Phylogeographic patterns of variation in birds tend to be longitudinal due to glaciation [16], [17]. Thus, genetic markers can complement information from analysis of hydrogen isotopes [11]. Putatively neutral genetic markers should present an unbiased estimate of population-level differences reflecting the interaction of gene flow and genetic drift only. Various genetic markers have been used in studies attempting to distinguish among breeding populations on the wintering grounds, with mitochondrial DNA (mtDNA) markers having been used most frequently [11], [18]–[22]. However, the patterns revealed by mtDNA are most often significant only at broad continental scales [11], [22]–[24]. Given their high variability and bi-parental inheritance, DNA microsatellite markers have proven useful for resolving population genetic questions at fine temporal and spatial scales [25], [26]. To date, microsatellites have not been used extensively in studies of migratory connectivity, perhaps in part because they have shown less genetic differentiation across populations than mtDNA in some bird species [23], [27]–[29]. Regardless of genetic marker, spatial genetic structure of breeding populations must first be quantified before probable origins of migratory individuals can be examined. Dispersal events may introduce biases as the genetic ‘signal’ is of natal origin rather than site of capture. The presence of “hybrids” between genetically differentiated populations will also complicate assignments.

Genetic and stable isotope markers have been used side-by-side in several studies of migratory connectivity to date, [11], [23], [30], [31]. Here, we extend this approach by using likelihoods from analysis of both isotopic and genetic markers combined probabilistically using Bayes Theorem to assign individuals to origin.

There are two major challenges in applying Bayesian approaches to assigning geographic origins to samples based upon stable-isotope analysis of feathers: choosing suitable prior probabilities (hereafter priors) and integrating these into Bayesian analysis. Various sources of information can be used to refine the assigned origins of unknown source birds based on isotopic data, and several methods have been applied [10], [12], [13], [32], [33]. Royle and Rubenstein [10] provided the first example of incorporating priors in a Bayesian assignment framework involving stable isotope analysis. They used data from two isotopes, with information from one isotope providing priors for the other. Data on species abundance derived from the Breeding Bird Survey (BBS) have been used as prior information for assignment of unknown origin birds to a source using stable isotopes in other studies [10], [13]. While these data are readily available, there are biases inherent in the BBS road-side based methodology; inadequate sampling in regions without roads, and problems associated with breeding phenology or detectability of species during the June census period [34]–[37]. In addition, the use of breeding ground abundance as a prior may bias assigned origins for juvenile birds if productivity does not show a strong correlation with breeding ground abundance [38]. More recently, Van Wilgenburg and Hobson [12] used analysis of banding recovery data as priors for analysis of data on stable hydrogen isotopic composition in feathers (hereafter δ^2^H_f_) to increase accuracy of assignment of birds moving through stopover sites. However, banding data can be limited for some species despite intensive mark and recapture schemes [5].

Given that genetic structuring is often longitudinal in northern temperate species in North America, with greater genetic distinction on east-west over north-south axes [27], [39], [40], and whereas δ^2^H_f_ isoscapes generally reflect broad latitudinal clines with little longitudinal variation [41], the combination of genetic and deuterium data in a Bayesian framework should improve assignment for birds of unknown origin on that continent. Our aim was to develop an approach to assignment based on genetic admixture coefficients of Loggerhead Shrike (*Lanius ludovicianus*) derived from spatial analysis of nuclear DNA microsatellites as priors for Bayesian assignment based on δ^2^H_f_. We use data from the Loggerhead Shrike to illustrate our novel approach. Results suggest that while both markers are capable of assigning individuals to their likely origin, our Bayesian approach was far superior to assessments based upon either genetic or δ^2^H_f_ alone.

## Materials and Methods

Adult shrikes were captured using a modified Potter trap [42] baited with a live mouse (*Mus mustellus*) in a protective wire ‘hardware-cloth’ cage. No mouse was used as bait in a trap for more than an hour at a time. While in the hardware-cloth cage, the mouse was provided with a slice of apple for nourishment. In between deployment, the live trap was placed out of the sun and covered with a breathable towel to reduce the stress level of the mouse. In between use, mice were housed in a mouse cage approximately 35 cm(l)×25 cm(w)×30 cm(h), with access to food and water *ad libitum*. To date, no mouse has been injured by a shrike or in handling.

**Figure 1:**
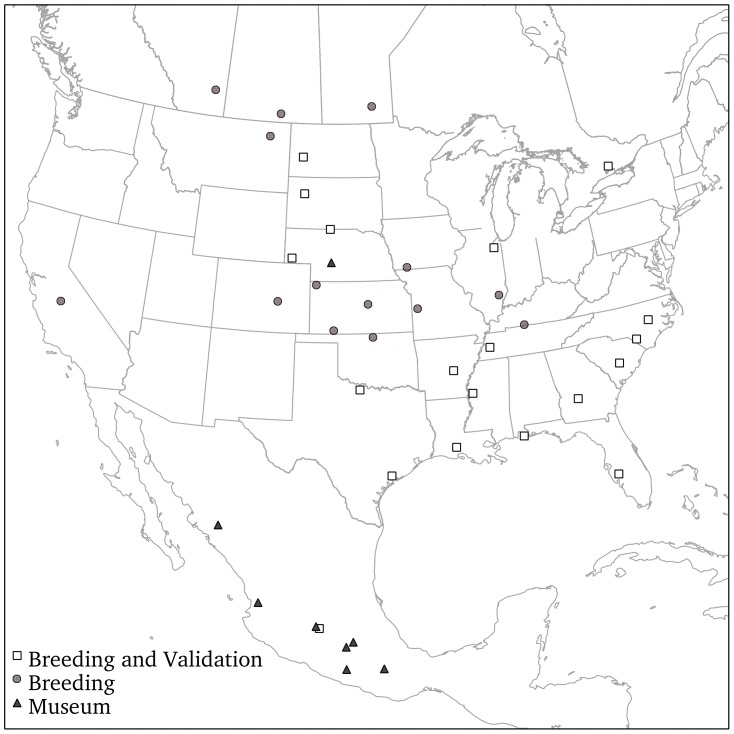
Sampling locales of Loggerhead Shrike feathers used for this study. A tail feather was obtained for DNA assays and a feather clipping of the first primary feather was obtained for stable hydrogen isotope analysis from each individual sampled to quantify genetic population structure and develop a species-specific isoscape, respectively. Areas in which samples were selected to test Bayesian methodology for assigning individuals to a probable origin using separate assignment analysis of microsatellite data as priors for analysis of δ^2^H_f_ data are noted as ‘breeding and validation’ areas.

Loggerhead Shrikes were sampled at 44 North American breeding locales across their geographic range during the breeding season (2004–2008) (Figure 1). Each bird was aged based on molt limits [43]. Following convention, we refer to any individual in the first year of life, starting from the time when they hatched (May-July) until the end of their first breeding season, when they undergo a complete molt [44]–[46] as being Second-Year (SY) and individuals in the second year of life or later as After Second-Year (ASY) birds.

Total genomic DNA was extracted from a ∼1 cm clipping of the distal tip of a plucked tail feather, using a QIAGEN (Venlo, Netherlands) DNEasy Extraction Kit as per Coxon et al. [47]. Fifteen microsatellite loci were assayed, including 14 primer pairs developed for use with Loggerhead Shrike [47], [48] and one developed for the Florida Scrub-Jay *Aphelocoma coerulescens* [49] but used previously for this species [48]. Genetic data were obtained from 767 of the shrikes sampled (Table S1). Genetic structure of individuals sampled during the breeding season was delineated using the Bayesian-clustering program STRUCTURE 2.3 [50]. Results from STRUCTURE [50] suggested five genetically and geographically distinct subpopulations. A detailed account of the methods and results of the analysis of genetic population structure can be found in Text S1.

The distal tip of P1 feather was clipped for use in stable isotope analysis; P1 is a feather that is almost exclusively grown on the breeding grounds in this species [45], [46]. Stable isotope (δ^2^H_f_) data were obtained from 556 of the shrikes sampled (Table S1). We added shrike feather samples from 40 museum specimens of known breeding provenance from across Mexico obtained from the Universidad Autónoma de Mexico to assist in calibration of the isoscape for Mexico. Measurements of δ^2^H_f_ were obtained using continuous-flow isotope-ratio mass spectrometry (CF-IRMS) following the ‘comparative equilibration’ technique described by Wassenaar and Hobson [51] at the stable-isotope facility of the National Water Research Centre in Saskatoon, Canada. These data were then used to derive a species-specific δ^2^H_f_ isoscape for SY and ASY individuals independently using the δ^2^H_f_ values of the feathers represented by our best sample of putatively known-origin birds. Methods used to derive our isoscapes are detailed in Text S1.

**Figure 2:**
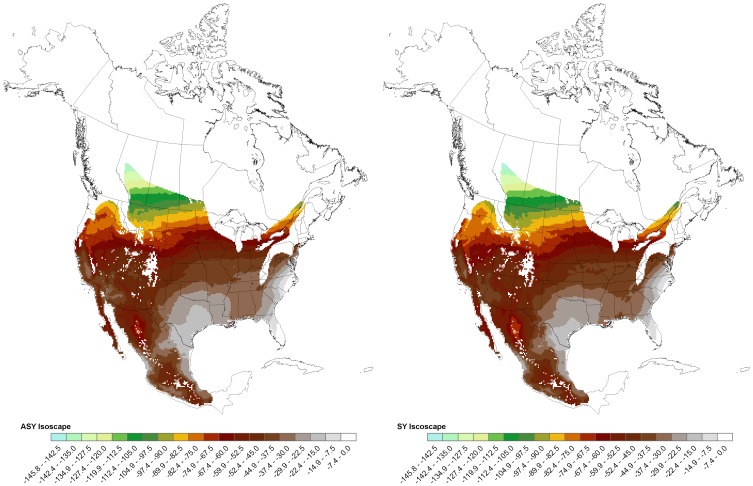
Stable hydrogen isoscapes for Second Year (SY) and After Second Year (ASY) aged Loggerhead Shrikes. Base map of expected δ^2^H_f_ values from Second Year (SY) and After Second Year (ASY) Loggerhead Shrikes derived from regression of δ^2^H_f_ against values predicted from a GIS-based model of expected growing season average δ^2^Hp in North America [24].

Using the data set for which we had both genetic and δ^2^H_f_ data (n = 515), we removed a subset of birds (n = 102, 32 SY and 70 ASY birds, 20% of the total in each age class) to assess the impact of the Bayesian approach on the study of connectivity (Table S1). Individuals in this subset (hereafter the validation samples) were chosen from 19 locales, representing a broad distribution of the species’ range (Figure 1). Validation samples were chosen randomly from sites with >6 individuals (Figure 2). Breeding ground origin was assigned to our validation samples based on genetic cluster using STRUCTURE [50]. Using only isotopic data, we assigned our validation samples to breeding-origin by determining the odds that any individual’s assigned geographic origin was correct by comparing the individual’s δ^2^H_f_ to that predicted by the age-specific δ^2^H_f_ isoscape. Specifically, based on 2∶1 odds that a given bird had truly originated from within the range defined by the odds ratio, we identified the set of raster cells that defined the upper 67% of estimated “probabilities of origin” and coded those as 1, and all others as 0, resulting in one binary map per individual. We also conducted analyses using 3∶1 odds, which identified the set of raster cells that defined the upper 75% of estimated probabilities of origin. The choice of odds ratio determines the compromise between risk of being incorrect and geographical resolution of probable breeding ground origin. We then used Bayes’ Theorem to compute the probability that a location was the origin of a bird given its δ^2^H_f_ value, conditional on the probability that the individual came from a given subpopulation given its *Q* genetic admixture coefficient from our STRUCTURE analysis.

To compare the probability densities modeled to describe potential origins using our Bayesian approach versus assignments based on δ^2^H_f_ alone, we calculated the total number of raster cells (equivalent to 20 km^2^ blocks) encompassed within the probable area of origin using both 2∶1 and 3∶1 odds ratios, with and without applying genetic priors. Assignment of the validation samples using deuterium feather values was undertaken using the ‘raster’ package [52]. Our isotopic assignment models were created using scripts written for the R statistical computing environment [53] by SVW. A detailed methods for the individual assignment to sample origin using the δ^2^H_f_ values, with and without priors, and with microsatellite data is provided in Text S1.

All sampling completed during this study complied with the current laws of the countries in which it was performed. Queen’s University’s Animal Care Committee approved the protocols used in sampling animals in this study (Protocol number Lougheed-2008-059-Or).

## Results

**Figure 3:**
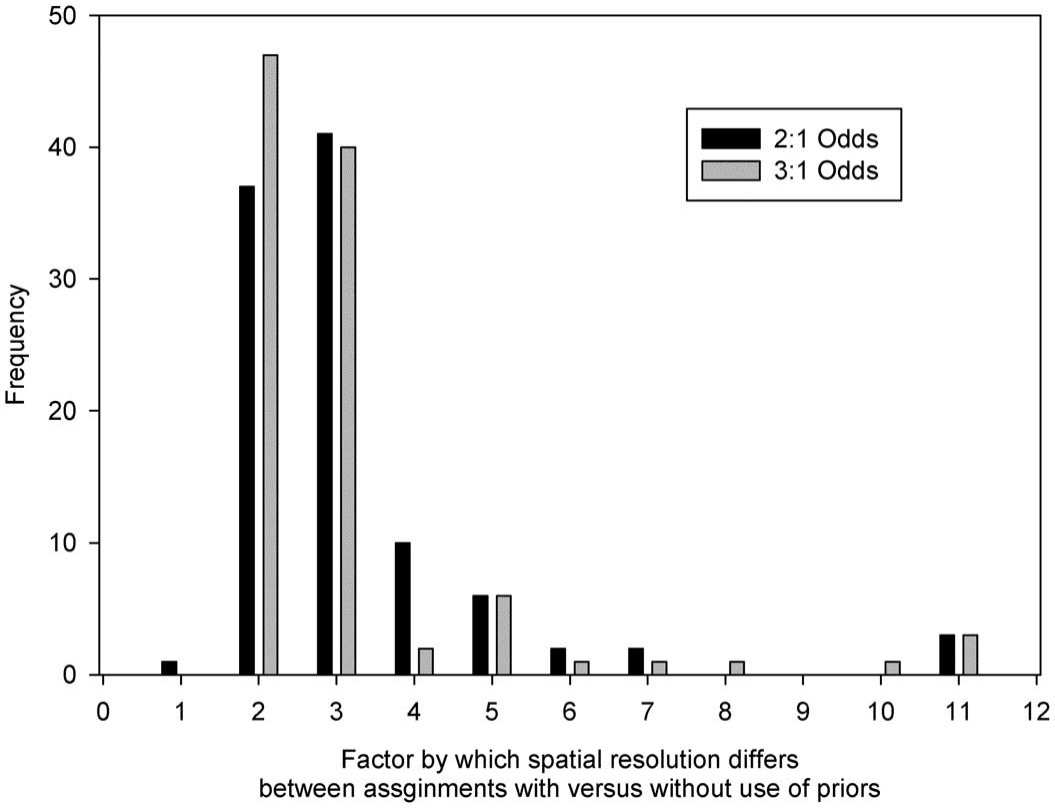
Factor by which spatial resolution differs between assignments with versus without the use of priors. Black bars represent number of individuals assigned using 2∶1 odds with priors. Grey bars represent number of individuals assigned using 3∶1 odds with priors.

The proportion of individuals in the validation sample set assigned to their putative origin remained essentially unchanged based on the δ^2^H_f_ isoscape versus genetic admixture coefficients alone with 71% of individuals assigned ‘correctly’ using microsatellite markers alone, 73% assigned correctly using the δ^2^H_f_ alone with 2∶1 odds ratio and 81% assigned correctly using the δ^2^H_f_ alone with 3∶1 odds ratio (Table S2). There was little difference in the size of the geographic area of likely origin for individuals based on 2∶1 versus 3∶1 odds ratios. The probable area of origin being somewhat larger using 3∶1 odds as expected (Table 1, Table S3), which affected the average and cumulative percentage of individuals assigned in the probable area of origin (Table S4). Our Bayesian models greatly reduced the probable area of origin over assignments based on δ^2^H_f_ alone – an average 2-fold decrease for SY birds and an average 3-fold decrease for ASY birds (Table 1, Figure 3).

**Table 1:** Average area assigned as probable area of origin for individuals in each sample area.

		2∶1 Odds Ratio		3∶1 Odds Ratio	
Sample locale	N	No Prior	Prior	No Prior	Prior
Alabama SY	2	6318±7383	1791±2065	7353±2065	2081±356
Alabama ASY	4	7686±171	1158±171	8853±201	1371±280
Arkansas SY	2	4636±3308	2805±1269	5397±3778	3393±1619
Arkansas ASY	4	6376±615	2868±615	7363±2265	3556±539
Florida SY	2	2149±570	1067±477	2533±645	1808±305
Florida ASY	4	1865±410	927±410	2244±889	1143±481
Georgia SY	2	6396±1474	3320±122	7389±1646	3766±139
Georgia ASY	4	6190±124	2658±124	7183±828	3123±85
Illinois N ASY	2	5609±532	1421±98	8247±602	1639±112
Louisiana SY	2	4082±535	2149±208	4855±621	2514±240
Louisiana ASY	4	5992±222	2031±222	6923±1494	2475±309
Michoacan SY	1	7800	3943	8974	4446
Michoacan ASY	1	7865	3302	9013	3826
Mississippi SY	2	5884±1190	3864±134	6889±1358	4541±262
Mississippi ASY	4	5916±521	3267±521	6852±1820	3917±596
Nebraska SY	2	4778±1154	1783±231	5706±1351	2290±281
Nebraska ASY	4	4504±82	1647±82	5317±1510	2167±167
North Carolina N SY	2	2543±1004	1211±350	3099±1179	1434±4055
North Carolina N ASY	4	1378±447	615±447	1660±1136	754±515
North Carolina S SY	2	1078±1121	493±355	1319±1287	619±434
North Carolina S ASY	4	1739±452	783±452	2102±1107	970±483
North Dakota SY	2	2186±887	2085±326	2534±1044	2386±425
North Dakota ASY	4	3209±230	2045±230	3682±436	2468±302
Ontario ASY	3	3538±466	286±30	4074±571	338±37
South Carolina SY	2	5950±765	2109±468	6704±1121	2372±460
South Carolina ASY	4	6039±99	1531±99	7039±1750	1784±107
South Dakota N SY	2	5001±1375	2820±592	5925±1581	3458±1361
South Dakota N ASY	4	4983±1228	2370±1228	5770±2114	3304±1484
South Dakota S SY	1	3169	1967	3705	2425
South Dakota S ASY	4	5891±1036	2423±1036	6859±1516	3072±1127
Tennessee SY	2	7024±230	2778±1131	8196±267	3568±1167
Tennessee ASY	4	6839±342	2172±342	7931±692	2752±452
Texas N SY	2	4592±3635	2045±808	5331±4204	2483±1126
Texas N ASY	4	3285±637	1414±637	3865±1237	1966±764
Texas SE SY	2	1476±427	1063±216	1826±510	1256±235
Texas SE ASY	4	3383±393	1601±393	3952±897	1962±469

Shown are the average (± standard deviation) number of raster cells (20 km^2^ blocks) assigned as the probable area of origin for each individual in the validation sample set using δ^2^H_f_ data alone and with Bayesian methodology using priors derived from analysis of microsatellite data for analysis of δ^2^H_f_ data.

**Figure 4:**
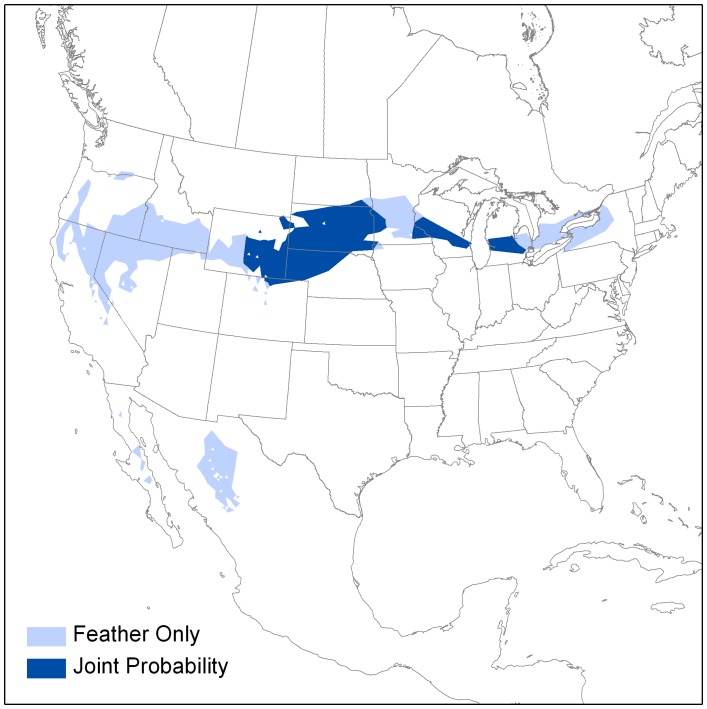
Example of an assignment of a single Loggerhead Shrike originating in South Dakota. Larger area shown in light blue encompass probable area of origin based only on the individual’s δ^2^H_f_ value, while reduced area shown in dark blue depicts probable area of origin based on the Bayesian methodology in which the individual’s *Q* genetic admixture coefficient is used as prior information in assignment based on the individual’s δ^2^H_f_.

On an individual basis, the decrease in spatial extent of the probable area of origin using our Bayesian priors was often even more pronounced, ranging up to a 13-fold decrease in the size of geographic area circumscribing (correctly) an individual ASY bird’s putative origin and a 5-fold decrease for individual SY birds (Figure 4, Table S3). The magnitude of the decrease varied by geographic locale, due both to the nature of the isoscape and the genetic characteristics of the region (Table 1, Figure 2, Table S3). Yet, even where the isocline (series of lines representing different δ^2^H values in the isoscape) was weak and *Q* genetic admixture coefficients were relatively low, the Bayesian methodology resulted in a much-reduced geographic area of probable origin (Table 1, Figure 2, Table S3).

## Discussion

We describe a Bayesian approach for assigning birds of unknown provenance to a breeding ground origin using both genetic and isotope data and validate our results using putatively ‘known source’ birds from across the species range in North America. We found dramatic improvement in the spatial resolution of the assigned area of origin using the Bayesian methodology with two types of intrinsic markers over models using δ^2^H_f_ or genetic data alone. The level of improvement depended on the interplay between the local steepness of the isotope isocline and the strength of genetic admixture coefficient *Q*.

### Bayesian Approach of Assessing Connectivity

Our Bayesian approach using informed priors resulted in improved resolution of the model-generated area of origin over the model using δ^2^H_f_ data alone for almost all validation samples and for all sample areas (Table 1, Figure 3, Table S3). In this regard, our results reflect those of other studies that have employed a Bayesian framework in studies of migratory connectivity [10], [12], [13], [32], [33]. However, previous studies have employed prior probabilities derived from extrinsic markers or breeding ground abundance, each with their own sources of bias [10], [12], [13], [32], [33]. In contrast, our method relies solely on intrinsic markers, and thus should be less prone to sample bias. In addition, our results highlight the power of the Bayesian approach in general [54]–[57].

The greatest improvement in resolving likely areas of origin using our Bayesian models occurred in areas with a steep isocline and high *Q* admixture coefficients, such as our South Carolina sample area (Q = 0.96 to 0.98) (Table 1, Table S3), but they also performed well where steep isoclines coincided with areas where individuals were assigned to a genetic cluster with low *Q* admixture coefficient values, such as in North Dakota (*Q* = 0.37 to 0.52) (Table 1, Table S3). However, our results suggest that the strength of an individual’s assignment to a genetic cluster (i.e. its *Q* value) also corresponded to the level of improved resolution, in particular in areas characterized by a weak isocline. For example, where the isocline was shallow and the *Q* admixture coefficients were low, as in our southeastern Texas sample locale (*Q* = 0.53 to 0.62), there was only a moderate decrease in the spatial extent of the likely geographic origin in the model using priors versus that using the δ^2^H_f_ data alone, but in areas where the isocline was relatively shallow but *Q* values were stronger, such as in our Louisiana (*Q* = 0.71 to 0.77) and northern Texas sample areas (*Q* = 0.74 to 0.83), the improvement in resolution was greater (Table 1, Table S3).

Our results from North Carolina and Nebraska (Table 1, Table S3), both characterized by steep isoclines, highlight another consideration of the use of genetic priors in our model. The spatial extent of a genetic cluster affects the resolution of the area assigned to be an individual’s probable origin. For example, the improvement in resolution using our Bayesian model was not as great for the birds from the sample locales in North Carolina, which are embedded within a broadly circumscribed genetic cluster, as it was for Nebraska samples, which are situated in a genetic cluster covering a smaller geographic area.

While the proportion of individuals assigned to their putative origin (i.e. sample area) remained essentially unchanged using the Bayesian model versus that based on a δ^2^H_f_ isoscape alone, this in part flows from real biological phenomena. Specifically, for philopatric birds, genetic diagnoses of origin will be concordant with those based on acquired markers, such as feather stable isotope values. However, assignments based on genetic and stable isotope data will be discordant for individuals that have dispersed beyond the boundaries of the spatial resolution for the isoscape. Thus, methodological improvements would presumably have little impact on rates of assignment. However, previously developed isoscapes [3], [38], [41], [58], [59] and results from studies of genetic population structure in birds [27], [39], [40] in North America often show non-random pattern, suggesting that a Bayesian methodology combining data from genetic and stable isotope markers should have broad applicability and be particularly powerful for improving the spatial resolution in studies of migratory connectivity.

### Impact of Dispersal on Assignment

Dispersal plays a fundamental role in determining range size and expansion [60]–[62], and in metapopulation [63] and source-sink dynamics [64]. As a pre-requisite to gene flow, dispersal rates among populations and the distribution of dispersal distances also relate to population genetic structure [65], [66]. Yet, dispersal remains a frontier for biological study [67] because it is difficult to track birds in consecutive seasons, or across large distances. Current techniques utilizing mark-recapture studies are biased toward the initial capture population [7]. Further, the upper limit of band recapture distance will necessarily be less than or equal to the size of the study area. Stable isotope data from inert tissues such as feathers provide a direct link between capture location and origin of molt (e.g. natal origin or previous breeding or wintering area) when patterns of molt have been described. Genetic markers are fixed from conception and thus provide information on the natal site of an individual, rather than simply its previous site of occupation. Previous work has shown that a multi-isotope approach can reliably delineate natal origins or molting locations of birds [68], [69] and nuclear DNA microsatellites can be used to estimate rates of immigration among populations [70] and infer dispersal distances [71]–[73]. Thus, intrinsic markers such as those used in this study, particularly when used in a Bayesian framework, may be especially useful in detecting and quantifying dispersal events.

We have presumed that the capture location of each individual during the breeding season was within its natal area, in the case of SY birds, or, in the case of ASY birds, its previous breeding area. Obviously, this will not be true for all samples as some first year breeders and older birds will disperse varying distances between breeding seasons [74]–[76]. We suggest that the apparent error in assignment in a small number of our samples is likely a result of dispersal, rather than model-based errors. The generally poorer rate of assignment of SY versus ASY shrikes in our study is consistent with previous work indicating a higher probability and longer dispersal distance in birds prior to breeding for the first time [76], [77]. Indeed, this apparent ‘error’ implies that our methodology may hold promise in detecting dispersers [78], [79].

The inclusion of short-distance dispersers when parameterizing our model would likely neither result in discordance between genetic and stable isotope data nor affect probability of assignment, unless isotopic or genetic spatial structure was pronounced. However, long-distance dispersal movements among breeding populations, specifically breeding season dispersal rather than migratory movements, would affect both model parameterization and assignment of individuals of unknown origin (e.g. migratory or wintering). While we did not specifically test for these effects, we suggest that the inclusion of isotopic data obtained from long-distance dispersers when parameterizing our model, regardless of where within the continental range they originated, would affect the resolution of our isocline, with a negative correlation between the number of long-distance dispersers and the strength of the isocline. Thus, a weakened isocline and increased variance in deuterium values would increase the putative area of origin and/or diminish the accuracy of assignment in assignments of unknown individuals to their geographic origins. Long-distance immigrants will be evident as isotopic outliers relative to others sampled within a locale. Deuterium values do vary locally and thus, while more stringent removal of such outliers would allow for finer-scale designation of origin for assigned individuals, it would also undoubtedly diminish the accuracy of results.

Long-distance dispersers also complicate analysis of genetic markers. STRUCTURE does not require a priori designation of sample populations, allowing immigrants to be assigned to their ‘correct’ (i.e. natal) genetic cluster, rather than to that of the genetic cluster representing the area in which they were sampled. The inclusion of immigrants diminishes resolution of the spatial genetic structuring. In population genetic terms, the number and natal origin (i.e. genetic heritage) of F1 hybrids – those individuals that result from successful dispersal and subsequent reproduction – will have a direct impact on the genetic structuring of the species. Dispersal can increase the extent of hybrid zones and result in loss of genetic structuring altogether. From the perspective of assignment of unknown individuals, the probability of assignment will be positively related to the strength of genetic structuring, the spatial resolution of genetic structure, and their own level of admixture.

### Future Directions

Methods for assignment of individuals to their putative breeding population origin have improved markedly since the isotopic method was first proposed for birds [80], [81]. Our results represent an important step in the study of migration biology, with implications for dispersal research, and provide insight into Bayesian assignment tests, but we also suggest that there may be a broader application for our data set and methodology. For example, we suggest that our methodology could be adapted for use with morphometric rather than genetic data as prior information, which may be a more cost-effective approach when species display distinct geography structure in morphology. Our Bayesian model could aid in the delineation of sources of productivity of migratory populations [38], [68], illuminating evolutionary processes [82], and aiding in tracking of disease [83], [84]. Coulton et al [69] showed that isotopic assignment models for waterfowl could be applied across species. Indeed, calibration algorithms linking δ^2^H_p_ with δ^2^H_f_ have shown general agreement across several avian species [12], [81], [84]–[86] but see Lott and Smith [56]. Thus our isoscape itself may facilitate conservation of a variety of migratory grassland and aridland species in North America, which, like the Loggerhead Shrike, are of conservation concern [79], [87]. If future testing shows that our model is more generally suitable, our isoscape would allow wildlife managers to measure connectivity among North American grassland bird populations in space and time without the need for extensive sampling ideally required for building species-specific models.

## Supporting Information

Table S1 **List of samples included in this study.** Given are the geographic coordinates for the range over which individuals were sampled in each area, number of samples for which nuclear microsatellite and deuterium data were available and number of samples used to validate methodology from each sample locale.
(DOCX)

Table S2 **Assignment of individuals in the validation sample set to origin using various models.** Shown is the number of individuals assigned to correct breeding ground origin based on nuclear microsatellite (Msat) data alone, and feather δ2H values using 2∶1 and 3∶1 odds ratios without priors.
(DOCX)

Table S3 **Probable area of origin for each individual in the validation sample set using various models.** Shown is the number of raster cells (20 km^2^ blocks) assigned as being the probable area of origin for each individual in each sample locale using δ^2^H_f_ data alone and using Bayesian methodology with nuclear microsatellite data as priors for deuterium data with 2∶1 and 3∶1 odds. Numbers in bold indicate individuals whose capture coordinates fell within the probable area of origin. Numbers in bold italics indicate individuals whose capture location fell within 200 km of the probable area of origin. Numbers in italics indicate individuals whose capture location fell within 400 km of the probable area of origin. Admixture coefficient corresponds to the genetic cluster with which the individual most closely assigned.
(DOCX)

Table S4 **Statistical comparison of expected versus observed assignment rates in the two Bayesian models.** Shown are the average number and cumulative percentage of individuals assigned in the probable area of origin or to within a buffer zone of varying size around the probable area of origin using δ^2^H_f_ data with 2∶1 and 3∶1 odds without priors. Bolded values signify tests where the number of correct assignments was greater than expected. χ^2^ tests performed with one degree of freedom.
(DOCX)

Text S1 (DOC)
