# Supplementary material for: Advances in Linking Wintering Migrant Birds to Their Breeding-Ground Origins Using Combined Analyses of Genetic and Stable Isotope Markers
Source: PLoS One. 2012 Aug 20;7(8):e43627. doi: 10.1371/journal.pone.0043627 (PMC3423384; doi:10.1371/journal.pone.0043627)
Supplement: Table S1 — List of samples included in this study. Given are the geographic coordinates for the range over which individuals were sampled in each area, number of samples for which nuclear microsatellite and deuterium data were available and number of samples used to validate methodology from each sample locale. (DOCX) [file pone.0043627.s001.docx]

**Table S1. List of samples included in this study.** Given are the geographic coordinates for the range over which individuals were sampled in each area, number of samples for which nuclear microsatellite and deuterium data were available and number of samples used to validate methodology from each sample locale.

| Sample locale | Latitude (°N) | Longitude (°W) | N_Msat_ | N_δ_^2^_H_ | N_Validators_ |
| --- | --- | --- | --- | --- | --- |
| Alabama, US | 30.32 to 30.41 | -87.68 to -87.77 | 25 | 25 | 6 |
| Alberta, CND | 49.10 to 51.48 | -110.00 to -112.07 | 52 | 0 | 0 |
| Arkansas, US | 34.45 to 34.78 | -91.87 to -92.17 | 22 | 22 | 6 |
| California, US | 33.50 to 38.67 | -117.00 to -122.00 | 39 | 0 | 0 |
| Coahuila N, MX | 25.28 to 25.38 | -100.61 to -101.42 | 0 | 3 | 0 |
| Colorado, US | 38.50 to 38.84 | -104.17 to -105.84 | 30 | 0 | 0 |
| Distrito Federal, MX | 19.20 | -99.09 | 0 | 3 | 0 |
| Durango, MX | 24.02 | -104.76 | 0 | 1 | 0 |
| Florida, US | 27.22 to 27.64 | -81.60 to -81.87 | 24 | 23 | 6 |
| Georgia W, US | 31.06 to 32.13 | -83.61 to -83.92 | 25 | 25 | 6 |
| Guanajuato, MX | 20.72 to 21.11 | -101.30 to -101.77 | 0 | 3 | 0 |
| Guerrero, MX | 17.55 | -99.50 | 0 | 2 | 0 |
| Illinois N, US | 41.35 to 41.40 | -88.03 to -88.17 | 10 | 34 | 2 |
| Illinois S, US | 38.61 to 38.73 | -87.89 to -88.19 | 11 | 11 | 0 |
| Iowa, US | 40.71 to 41.43 | -94.37 to -95.17 | 8 | 0 | 0 |
| Kansas-C, US | 39.38 to 39.84 | -101.54 to -101.77 | 6 | 0 | 0 |
| Kansas-NW, US | 38.61 to 38.67 | -97.76 to -97.94 | 0 | 0 | 0 |
| Kansas-SW, US | 37.01 to 37.10 | -100.11 to -100.36 | 8 | 5 | 0 |
| Kentucky, US | 36.56 to 36.66 | -86.59 to -86.78 | 10 | 10 | 0 |
| Louisiana, US | 30.04 to 30.25 | -92.09 to -92.34 | 23 | 23 | 6 |
| Manitoba, CND | 49.67 to 50.15 | -96.75 to -97.65 | 41 | 0 | 0 |
| Mexico State, MX | 18.90 to 19.09 | -99.50 to -100.07 | 0 | 3 | 0 |
| Michoacan, MX | 19.47 to 20.01 | -101.09 to -101.60 | 24 | 16 | 2 |
| Mississippi, US | 33.13 to 33.22 | -90.75 to -91.03 | 25 | 25 | 6 |
| Missouri, US | 38.13 to 38.29 | -94.16 to -94.33 | 11 | 0 | 0 |
| Montana, US | 47.42 to 48.24 | -104.34 to -105.78 | 10 | 10 | 0 |
| Morelos, MX | 18.70 to 18.86 | -98.98 to -99.25 | 0 | 4 | 0 |
| Nayarit, MX | 21.39 to 21.55 | -104.83 to -104.86 | 0 | 3 | 0 |
| Nebraska, US | 41.17 to 42.93 | -100.50 to -103.67 | 21 | 21 | 6 |
| North Carolina N, US | 35.32 to 35.82 | -77.09 to -77.91 | 25 | 25 | 6 |
| North Carolina S, US | 34.41 to 34.67 | -78.97 to -79.20 | 25 | 25 | 6 |
| North Dakota, US | 46.26 to 47.42 | -102.63 to -103.36 | 20 | 18 | 6 |
| Nuevo Leon E, MX | 24.71 | -100.23 | 0 | 1 | 0 |
| Oaxaca, MX | 17.55 to 17.80 | -97.28 to -97.77 | 0 | 2 | 0 |
| Oklahoma N, US | 36.23 to 36.97 | -95.78 to -99.25 | 11 | 11 | 0 |
| Ontario, CND | 44.32 to 45.47 | -76.65 to -81.13 | 34 | 72 | 3 |
| Saskatchewan, CND | 49.10 to 51.94 | -101.91 to -109.81 | 94 | 0 | 0 |
| Sinaloa, MX | 25.73 | -107.51 | 0 | 1 | 0 |
| South Carolina, US | 33.36 to 33.67 | -80.20 to -80.92 | 25 | 25 | 6 |
| South Dakota N, US | 44.36 to 45.20 | -102.28 to -103.13 | 21 | 20 | 6 |
| South Dakota S, US | 43.03 to 43.20 | -100.11 to -100.75 | 15 | 14 | 5 |
| Tennessee W, US | 35.63 to 35.99 | -88.94 to -89.32 | 24 | 23 | 6 |
| Texas N, US | 33.66 to 33.87 | -98.22 to -98.64 | 22 | 22 | 6 |
| Texas SE, US | 28.66 to 28.98 | -96.41 to -96.68 | 26 | 25 | 6 |
