# Supplementary material for: Advances in Linking Wintering Migrant Birds to Their Breeding-Ground Origins Using Combined Analyses of Genetic and Stable Isotope Markers
Source: PLoS One. 2012 Aug 20;7(8):e43627. doi: 10.1371/journal.pone.0043627 (PMC3423384; doi:10.1371/journal.pone.0043627)
Supplement: Table S2 — Assignment of individuals in the validation sample set to origin using various models. Shown is the number of individuals assigned to correct breeding ground origin based on nuclear microsatellite (Msat) data alone, and feather δ2H values using 2∶1 and 3∶1 odds ratios without priors. (DOCX) [file pone.0043627.s002.docx]

**Table S2. Assignment of individuals in the validation sample set to origin using various models.** Shown is the number of individuals assigned to correct breeding ground origin based on nuclear microsatellite (Msat) data alone, and feather δ2H values using 2:1 and 3:1 odds ratios without priors.

|  |  |  | 2:1 Odds Ratio | 3:1 Odds Ratio |
| --- | --- | --- | --- | --- |
| Sample locale | N | Msat | No Prior | No Prior |
| Alabama SY | 2 | 2 | 0 | 2 |
| Alabama ASY | 4 | 4 | 0 | 0 |
| Arkansas SY | 2 | 0 | 0 | 1 |
| Arkansas ASY | 4 | 0 | 3 | 3 |
| Florida SY | 2 | 0 | 2 | 2 |
| Florida ASY | 4 | 4 | 4 | 4 |
| Georgia SY | 2 | 1 | 2 | 2 |
| Georgia ASY | 4 | 1 | 4 | 4 |
| Illinois N ASY | 2 | 2 | 0 | 1 |
| Louisiana SY | 2 | 2 | 2 | 2 |
| Louisiana ASY | 4 | 4 | 4 | 4 |
| Michoacan SY | 1 | 1 | 0 | 1 |
| Michoacan ASY | 1 | 1 | 1 | 1 |
| Mississippi SY | 2 | 0 | 1 | 1 |
| Mississippi ASY | 4 | 0 | 4 | 4 |
| Nebraska SY | 2 | 2 | 2 | 2 |
| Nebraska ASY | 4 | 4 | 3 | 3 |
| North Carolina N SY | 2 | 2 | 1 | 2 |
| North Carolina N ASY | 4 | 4 | 4 | 4 |
| North Carolina S SY | 2 | 2 | 2 | 2 |
| North Carolina S ASY | 4 | 4 | 4 | 4 |
| North Dakota SY | 2 | 0 | 1 | 1 |
| North Dakota ASY | 4 | 0 | 4 | 4 |
| Ontario ASY | 3 | 3 | 3 | 3 |
| South Carolina SY | 2 | 2 | 2 | 2 |
| South Carolina ASY | 4 | 4 | 2 | 2 |
| South Dakota N SY | 2 | 2 | 0 | 0 |
| South Dakota N ASY | 4 | 4 | 2 | 3 |
| South Dakota S SY | 1 | 1 | 0 | 0 |
| South Dakota S ASY | 4 | 2 | 2 | 3 |
| Tennessee SY | 2 | 1 | 2 | 2 |
| Tennessee ASY | 4 | 4 | 4 | 4 |
| Texas N SY | 2 | 2 | 0 | 1 |
| Texas N ASY | 4 | 4 | 3 | 3 |
| Texas SE SY | 2 | 1 | 2 | 2 |
| Texas SE ASY | 4 | 2 | 4 | 4 |
