# Supplementary material for: Advances in Linking Wintering Migrant Birds to Their Breeding-Ground Origins Using Combined Analyses of Genetic and Stable Isotope Markers
Source: PLoS One. 2012 Aug 20;7(8):e43627. doi: 10.1371/journal.pone.0043627 (PMC3423384; doi:10.1371/journal.pone.0043627)
Supplement: Table S3 — Probable area of origin for each individual in the validation sample set using various models. Shown is the number of raster cells (20 km2 blocks) assigned as being the probable area of origin for each individual in each sample locale using δ2Hf data alone and using Bayesian methodology with nuclear microsatellite data as priors for deuterium data with 2∶1 and 3∶1 odds. Numbers in bold indicate individuals whose capture coordinates fell within the probable area of origin. Numbers in bold italics indicate individuals whose capture location fell within 200 km of the probable area of origin. Numbers in italics indicate individuals whose capture location fell within 400 km of the probable area of origin. Admixture coefficient corresponds to the genetic cluster with which the individual most closely assigned. (DOCX) [file pone.0043627.s003.docx]

**Table S3. Probable area of origin for each individual in the validation sample set using various models.** Shown is the number of raster cells (20km^2^ blocks) assigned as being the probable area of origin for each individual in each sample locale using δ^2^H_f_ data alone and using Bayesian methodology with nuclear microsatellite data as priors for deuterium data with 2:1 and 3:1 odds. Numbers in bold indicate individuals whose capture coordinates fell within the probable area of origin. Numbers in bold italics indicate individuals whose capture location fell within 200 km of the probable area of origin. Numbers in italics indicate individuals whose capture location fell within 400 km of the probable area of origin. Admixture coefficient corresponds to the genetic cluster with which the individual most closely assigned.

|  |  |  | Admixture | 2:1 Odds Ratio | | 3:1 Odds Ratio | |
| --- | --- | --- | --- | --- | --- | --- | --- |
| Sample locale | Age | δ^2^H_f_ | Coefficient | No Prior | Prior | No Prior | Prior |
| Alabama | SY | -27 | 0.96 | ***5009*** | ***2062*** | **5893** | ***2333*** |
| Alabama | SY | -51 | 0.94 | 7626 | **1520** | **8813** | **1829** |
| Alabama | ASY | -51 | 0.97 | 7540 | **1056** | 8706 | **1391** |
| Alabama | ASY | -51 | 0.97 | 7502 | **979** | 8671 | **1246** |
| Alabama | ASY | -49 | 0.93 | 7778 | **1246** | 8940 | **1750** |
| Alabama | ASY | -47 | 0.95 | 7923 | **1352** | 9095 | **1097** |
| Arkansas | SY | -90 | 0.57 | 2297 | 1907 | **2725** | 2248 |
| Arkansas | SY | -36 | 0.44 | 6975 | 3702 | 8068 | 4537 |
| Arkansas | ASY | -18 | 0.67 | **3389** | **2507** | **4055** | **3070** |
| Arkansas | ASY | -31 | 0.43 | **6860** | **3450** | **7898** | **4112** |
| Arkansas | ASY | -33 | 0.46 | **7255** | **3324** | **8348** | **3923** |
| Arkansas | ASY | -41 | 0.71 | ***8001*** | **2191** | ***9151*** | **3118** |
| Florida | SY | -5 | 0.50 | **1746** | **730** | **2077** | **1592** |
| Florida | SY | -12 | 0.56 | **2552** | **1404** | **2989** | **2023** |
| Florida | ASY | 2 | 0.71 | **983** | **460** | **1226** | **621** |
| Florida | ASY | -6 | 0.69 | **1719** | **864** | **2103** | **1048** |
| Florida | ASY | -7 | 0.68 | **1859** | **925** | **2258** | **1117** |
| Florida | ASY | -14 | 0.60 | **2863** | **1458** | **3390** | **1784** |
| Georgia | SY | -27 | 0.50 | **5353** | **3234** | **6225** | **3667** |
| Georgia | SY | -37 | 0.60 | **7438** | **3406** | **8553** | **3864** |
| Georgia | ASY | -26 | 0.53 | **5442** | *2662* | **6329** | *3095* |
| Georgia | ASY | -27 | 0.53 | **5698** | **2715** | **6630** | **3162** |
| Georgia | ASY | -32 | 0.66 | **6706** | **2484** | **7763** | **3020** |
| Georgia | ASY | -34 | 0.54 | **6914** | **2770** | **8010** | **3216** |
| Illinois N | ASY | -33 | 0.94 | ***6761*** | ***1352*** | ***7821*** | ***1559*** |
| Illinois N | ASY | -39 | 0.93 | ***7513*** | ***1490*** | **8672** | ***1718*** |
| Louisiana | SY | -22 | 0.73 | **3703** | **2002** | **4416** | **2344** |
| Louisiana | SY | -25 | 0.71 | **4460** | **1729** | **4925** | **2683** |
| Louisiana | ASY | -20 | 0.75 | **4190** | **1729** | **4925** | **2098** |
| Louisiana | ASY | -26 | 0.77 | **5758** | **2076** | **6640** | **2421** |
| Louisiana | ASY | -31 | 0.70 | **6912** | **2264** | **7952** | **2847** |
| Louisiana | ASY | -32 | 0.76 | **7107** | **2054** | **8173** | **2534** |
| Michoacan | SY | -46 | 0.98 | ***7800*** | ***3943*** | **8974** | ***4446*** |
| Michoacan | ASY | -48 | 0.97 | **7865** | **3302** | **9013** | **3826** |
| Mississippi | SY | -28 | 0.35 | **5042** | **3769** | **5928** | **4355** |
| Mississippi | SY | -59 | 0.38 | 6725 | 3958 | **7849** | 4726 |
| Mississippi | ASY | -20 | 0.47 | **4210** | **2617** | **4948** | **3209** |
| Mississippi | ASY | -22 | 0.48 | **4852** | **3155** | **5656** | **3751** |
| Mississippi | ASY | -33 | 0.42 | **7146** | **3436** | **8213** | **4076** |
| Mississippi | ASY | -35 | 0.40 | **7457** | **3859** | **8591** | **4631** |
| Nebraska | SY | -74 | 0.69 | **3962** | **1946** | **4750** | **2489** |
| Nebraska | SY | -65 | 0.83 | **5594** | **1620** | **6661** | **2091** |
| Nebraska | ASY | -83 | 0.80 | *2755* | ***1682*** | *3199* | ***2123*** |
| Nebraska | ASY | -68 | 0.73 | **4475** | **1656** | **5292** | **2207** |
| Nebraska | ASY | -64 | 0.80 | **5245** | **1531** | **6219** | **1968** |
| Nebraska | ASY | -62 | 0.78 | **5541** | **1719** | **6557** | **2369** |
| North Carolina N | SY | -12 | 0.97 | **1833** | **963** | **2265** | **1147** |
| North Carolina N | SY | -21 | 0.95 | ***3253*** | ***1458*** | **3933** | ***1720*** |
| North Carolina N | ASY | 21 | 0.97 | **394** | **104** | **436** | **142** |
| North Carolina N | ASY | 1 | 0.97 | **1037** | **495** | **1304** | **641** |
| North Carolina N | ASY | -3 | 0.94 | **1410** | **678** | **1745** | **844** |
| North Carolina N | ASY | -13 | 0.90 | **2670** | **1181** | **3156** | **1387** |
| North Carolina S | SY | 6 | 0.90 | **285** | **242** | **4009** | **312** |
| North Carolina S | SY | -6 | 0.66 | **1870** | ***363*** | **1088** | **523** |
| North Carolina S | ASY | 3 | 0.94 | **836** | **363** | **1088** | **523** |
| North Carolina S | ASY | 2 | 0.92 | **973** | **426** | **1216** | **589** |
| North Carolina S | ASY | -11 | 0.92 | **2428** | **1110** | **2880** | **1313** |
| North Carolina S | ASY | -13 | 0.89 | **2720** | **1232** | **3225** | **1456** |
| North Dakota | SY | -106 | 0.45 | ***1559*** | *1854* | ***1796*** | ***2085*** |
| North Dakota | SY | -87 | 0.51 | **2813** | **2315** | **3272** | **2686** |
| North Dakota | ASY | -87 | 0.47 | **2852** | **1741** | **3258** | **2083** |
| North Dakota | ASY | -86 | 0.37 | **2933** | **2030** | **3352** | **2441** |
| North Dakota | ASY | -79 | 0.52 | **3527** | **2116** | **4062** | **2534** |
| North Dakota | ASY | -79 | 0.45 | **3523** | **2292** | **4054** | **2815** |
| Ontario | ASY | -83 | 0.99 | **3172** | **257** | **3624** | **298** |
| Ontario | ASY | -80 | 0.97 | **3380** | **283** | **3882** | **346** |
| Ontario | ASY | -74 | 0.99 | **4063** | **317** | **4717** | **371** |
| South Carolina | SY | -27 | 0.96 | **5409** | **1778** | **5911** | **2047** |
| South Carolina | SY | -32 | 0.97 | **6491** | **2440** | **7496** | **2697** |
| South Carolina | ASY | -19 | 0.96 | **3749** | *1508* | **4475** | *1784* |
| South Carolina | ASY | -30 | 0.98 | **6361** | **1668** | **7391** | **1928** |
| South Carolina | ASY | -34 | 0.98 | *6901* | ***1513*** | *7994* | **1753** |
| South Carolina | ASY | -36 | 0.97 | *7146* | **1433** | *8297* | **1672** |
| South Dakota N | SY | -23 | 0.68 | 4029 | 2401 | 4811 | 2495 |
| South Dakota N | SY | -64 | 0.75 | ***5973*** | **3238** | ***7047*** | **4420** |
| South Dakota N | ASY | -80 | 0.72 | **3413** | **1751** | **3915** | **2353** |
| South Dakota N | ASY | -79 | 0.77 | **3470** | **1292** | **3984** | **2004** |
| South Dakota N | ASY | -60 | 0.61 | 6275 | **4096** | **7327** | **5289** |
| South Dakota N | ASY | -57 | 0.83 | *6775* | **2340** | *7855* | **3568** |
| South Dakota S | SY | -83 | 0.79 | ***3169*** | **1967** | ***3705*** | **2425** |
| South Dakota S | ASY | -70 | 0.75 | **4520** | **1502** | **5278** | **2019** |
| South Dakota S | ASY | -67 | 0.59 | **5153** | **2312** | **6037** | **2830** |
| South Dakota S | ASY | -60 | 0.79 | ***6352*** | **1981** | **7416** | **2770** |
| South Dakota S | ASY | -51 | 0.58 | ***7540*** | ***3895*** | ***8706*** | **4670** |
| Tennessee | SY | -38 | 0.52 | **6861** | **3578** | **8007** | **4393** |
| Tennessee | SY | -41 | 0.75 | **7186** | **1978** | **8385** | **2743** |
| Tennessee | ASY | -29 | 0.80 | **6148** | **1703** | **7149** | **2106** |
| Tennessee | ASY | -31 | 0.72 | **6532** | **2402** | **7572** | **2993** |
| Tennessee | ASY | -36 | 0.67 | **7187** | **2449** | **8352** | **3120** |
| Tennessee | ASY | -39 | 0.70 | **7490** | **2134** | **8651** | **2790** |
| Texas N | SY | -98 | 0.83 | 2021 | 1473 | 2358 | 1687 |
| Texas N | SY | -38 | 0.81 | ***7162*** | **2616** | **8304** | **3279** |
| Texas N | ASY | -6 | 0.82 | *2066* | **1025** | *2444* | **1337** |
| Texas N | ASY | -15 | 0.85 | **3307** | **864** | **3835** | **1483** |
| Texas N | ASY | -16 | 0.77 | **3142** | **1479** | **3719** | **2019** |
| Texas N | ASY | -23 | 0.74 | **4625** | **2286** | **5461** | **3023** |
| Texas SE | SY | -3 | 0.53 | **1174** | *910* | **1465** | ***1089*** |
| Texas SE | SY | -9 | 0.61 | **1778** | ***1215*** | **2186** | ***1422*** |
| Texas SE | ASY | -12 | 0.53 | **2878** | **1402** | **3351** | **1744** |
| Texas SE | ASY | -13 | 0.69 | **2984** | **1289** | **3462** | **1557** |
| Texas SE | ASY | -14 | 0.56 | **3196** | **1543** | **3718** | **1916** |
| Texas SE | ASY | -22 | 0.63 | **4474** | **2169** | **5278** | **2630** |
