# Supplementary material for: Advances in Linking Wintering Migrant Birds to Their Breeding-Ground Origins Using Combined Analyses of Genetic and Stable Isotope Markers
Source: PLoS One. 2012 Aug 20;7(8):e43627. doi: 10.1371/journal.pone.0043627 (PMC3423384; doi:10.1371/journal.pone.0043627)
Supplement: Table S4 — Statistical comparison of expected versus observed assignment rates in the two Bayesian models. Shown are the average number and cumulative percentage of individuals assigned in the probable area of origin or to within a buffer zone of varying size around the probable area of origin using δ2Hf data with 2∶1 and 3∶1 odds without priors. Bolded values signify tests where the number of correct assignments was greater than expected. χ2 tests performed with one degree of freedom. (DOCX) [file pone.0043627.s004.docx]

**Table S4. Statistical comparison of expected versus observed assignment rates in the two Bayesian models.** Shown are the average number and cumulative percentage of individuals assigned in the probable area of origin or to within a buffer zone of varying size around the probable area of origin using δ^2^H_f_ data with 2:1 and 3:1 odds without priors. Bolded values signify tests where the number of correct assignments was greater than expected. χ^2^ tests performed with one degree of freedom.

|  | 2:1 Odds No Priors | | | | | | | | 3:1 Odds No Priors | | | | | | | |
| --- | --- | --- | --- | --- | --- | --- | --- | --- | --- | --- | --- | --- | --- | --- | --- | --- |
|  | SY | | | | ASY | | | | SY | | | | ASY | | | |
| Assignment | # | % | χ^2^ | p | # | % | χ^2^ | p | # | % | χ^2^ | p | # | % | χ^2^ | p |
| In area | 20 | 63 | 0.12 | 0.73 | 56 | 80 | 3.18 | 0.07 | 27 | 84 | 0.87 | 0.35 | 58 | 83 | 1.30 | 0.25 |
| 100 km | 25 | 78 | 1.06 | 0.30 | 57 | 81 | **3.97** | **0.04** | 29 | 91 | 2.74 | 0.10 | 59 | 84 | 1.86 | 0.17 |
| 200 km | 27 | 84 | 1.96 | 0.16 | 61 | 83 | **8.26** | **<0.005** | 30 | 94 | **4.27** | **0.04** | 61 | 87 | 3.36 | 0.07 |
| 400 km | 27 | 84 | 1.96 | 0.16 | 65 | 87 | **14.87** | **<0.001** | 30 | 94 | **4.27** | **0.04** | 66 | 94 | **10.01** | **<0.005** |
| 2000 km | 32 | 100 | **12.81** | **<0.001** | 70 | 100 | **28.00** | **<0.001** | 32 | 100 | **9.15** | **<0.001** | 70 | 100 | **20.00** | **<0.001** |
