# Supplementary material for: Advances in Linking Wintering Migrant Birds to Their Breeding-Ground Origins Using Combined Analyses of Genetic and Stable Isotope Markers
Source: PLoS One. 2012 Aug 20;7(8):e43627. doi: 10.1371/journal.pone.0043627 (PMC3423384; doi:10.1371/journal.pone.0043627)
Supplement: Text S1 — (DOC) [file pone.0043627.s005.doc]

**Supporting Methods & Results.**

The following summarizes methodology for quantifying genetic population structure in the Loggerhead Shrike, delineation of species- and age-specific isotopic maps, and results of assignment of samples to probable origin using a single marker approach.

*Molecular Genetic Methods and Analyses*

Total genomic DNA was extracted from an ~1cm clipping of the proximal tip of a plucked tail feather, using a QIAGEN (Venlo, Netherlands) DNEasy Extraction Kit as per Coxon et al. [1]. Fifteen microsatellite loci were assayed, including 14 primer pairs developed for use with Loggerhead Shrike [1,2] and one developed for the Florida Scrub-Jay *Aphelocoma coerulescens* [3] but used previously for this species [2]. The thermal cycling profiles were as per Coxon et al. [1]. PCR products from 6 loci (Llu40, LLU89, Llu133, LS4 and SJR4) were genotyped using a Li-Cor DNA sequencer (IR2 System), with scoring of each individual’s genotype done manually using IRD 700 50-350 base pair size standards. PCR products from an additional 11 loci (Llu15, Llu39, Llu55, Llu82, Llu85, Llu90, Llu95, Llu102, Llu112, Llu176) were genotyped using a Beckman Coulter CEQ8000 capillary automated sequencer, with alleles scored using the CEQ8000 Genetic Analysis System.

Analysis of genetic structure of individuals sampled during the breeding season was undertaken using the Bayesian-clustering programs STRUCTURE 2.3 [4] and TESS 2.1 [5]. These two programs use different algorithms based on different assumptions of the data. By comparing results from these programs we increased our confidence in the derived value of *k* (the number of unique genetic clusters or groupings). STRUCTURE combines information from multiple loci into a single probability model, and estimates the amount of genetic partitioning among individuals and populations [4]. Individuals are assigned to one or more genetic clusters based upon their level of admixture, as designated by the admixture coefficient (*Q*) [4,6]. TESS uses a spatial assignment approach to group individuals into genetic clusters, each of which is in Hardy Weinberg Equilibrium, and assumes geographical continuity of allele frequencies by including an interaction parameter. Thus, samples that are spatially close in the connection network are given a higher probability of belonging to the same genetic cluster than samples that are further apart [5]. Neither program requires *a priori* population designations, allowing us to designate genetic clusters *post hoc* based on genetic distinctiveness of individuals among sample areas.

STRUCTURE was run 20 times for each value of *k* from 1 to 20, using the admixture model with correlated allele frequencies [7]. A burn-in period of 100,000 replicates and 1,000,000 MCMC iterations was selected based on the values of the summary statistics output by the program in test runs, as suggested by Pritchard et al. [4]. The best model was selected by determining when the posterior probability values of *k* reached a plateau [4], and using an *ad hoc* statistic based on the rate of change between runs for successive values of *k* [8]. TESS was run 20 times for each value of k from 2 to 20 using the conditional autoregressive (CAR) admixture model [7], a burn-in of 20,000 replicates and 50,000 iterations based on output from initial test runs. The CAR model was chosen because geographical sampling was done discretely across the sample range. The number of clusters was determined by assessing when the deviance information criterion (DIC) values reached a plateau and the *Q*-matrix stabilized [5].

To control for between-run variation from TESS and STRUCTURE, the output from all 20 runs for the chosen best *k* was exported to CLUMPP [9]. We used the LargeK-Greedy algorithm, with 30,000 random input orders and H as the estimate of pairwise similarity between matrices [9].

Using output from CLUMPP [9], we assigned each individual to the genetic group for which it had the highest mean membership coefficient. Comparison of assignments of individuals to a genetic cluster based on their admixture coefficients revealed an 81.40% agreement in individual assignments between programs. Thus, we used only results from STRUCTURE for subsequent analyses. The spatial structuring of the genetic clusters was visualized by exporting the mean membership coefficients output from STRUCTURE to ArcGIS™ (ESRI, Redlands, CA). We used an ordinary point kriging technique, where values of unsampled areas are interpolated using data from known localities, using an exponential model.

Once breeding population genetic structure was defined, we assigned a breeding ground origin to our validation samples using STRUCTURE [4]. Recognizing that in genetic clusters we may have sampled individuals of mixed ancestry, we compiled a data set including only those breeding individuals that assigned with 80% or greater probability to a genetic group [10-12]. This data set was then combined for use as ‘trainers’ with the validation samples using “Prior Population Information to Assess Migrants” model in STRUCTURE [4]. The model was run with correlated allele frequencies, a burn-in period of 100,000 replicates and 1,000,000 MCMC iterations. The genetic admixture coefficients of winter-origin individuals were averaged across 20 runs in CLUMPP [9] using the parameters noted above. In this case, individuals were assigned to a genetic cluster (i.e. breeding ground origin) if they had a 0.60 or greater admixture coefficient in a group. We reasoned that if more than half the genome of an individual assigned to a genetic group then they could be assigned with some confidence [10]. Spatial structure of each of the genetic groups in the wintering season was visualized using individual admixture coefficients in ArcGIS™ (ESRI, Redlands, CA).

*Stable Isotope Methods and Analyses*

An approximately 1.0 x 0.5 cm section of the distal tip of the first primary (P1) feather was clipped for use in stable isotope analysis; P1 is a feather that is almost exclusively grown on the breeding grounds in this species [13,14]. We added feather tissue from 40 museum specimens of shrikes of known breeding provenance from across Mexico obtained using specimens kept at the Universidad Autónoma de Mexico to assist in calibration of the isoscape for Mexico (see below). Feathers were cleaned in 2:1 chloroform:methanol solution overnight, drained, and air dried in a fumehood. Feather tissue was then cut and 350 μg ± 10 μg weighed into 4.0 x 3.2 mm silver capsules for online hydrogen isotope analysis by continuous-flow isotope-ratio mass spectrometry (CF-IRMS). Stable hydrogen isotope analytical measurements (δ^2^H) followed the ‘comparative equilibration’ technique described by Wassenaar and Hobson [15]. This method includes pre-calibrated keratin working standards run along with unknown tissue samples, which are then isolated from the atmosphere using a zero-blank autosampler and analyzed in a single session, thus ensuring quality and accuracy of results [16]. External repeatability was estimated to be  ±3.2 ‰ based upon the long-term distribution of within-autorun residuals of accepted versus measured values from repeated analyses of hydrogen isotope inter-comparison material IAEA-CH-7 (-100 ‰) and our calibrated keratin reference materials (CHS,CFS,BWB).

Stable hydrogen isotope ratios are reported as the non-exchangeable hydrogen expressed in the typical delta notation in units of per mil (‰) normalized on the Vienna Standard Mean Ocean Water-Standard Light Antarctic Precipitation (VSMOW-SLAP) scale. The overall δ^2^H variance in keratinous tissues (e.g. hair or feathers) that is associated with internal metabolic processes and laboratory methodology is of the order of ±3‰ [17,18], which is very close to the analytical error inherent in CF-IRMS measurements for δ^2^H (± 2‰) [16]. Feather samples were analyzed at the stable-isotope facility of the National Water Research Centre in Saskatoon, Canada.

Despite the success in using δ^2^H values in feather tissue to assign origins to wintering birds [19-23], analytical error, within population inter-individual variance in δD feather values (hereafter δ^2^H_f_) and errors associated with depiction of an isoscape influence the accuracy of assignment [24,25]. Within-population variance in feather δ^2^H values can be influenced by age effects [26], dispersal among populations [27] and species effects [26]. Regional variation in δ^2^H precipitation values (hereafter δ^2^H_p_) also affects accuracy of assignment [26]. We investigated and attempted to incorporate variance due to these factors in our isoscape model as follows.

We derived a species-specific δ^2^H_f_ isoscape [23,26]. To convert the amount-weighted mean growing-season precipitation δ^2^H isoscape (δ^2^H_p_) of Bowen et al. [28] to an expected δ^2^H_f_ isoscape by regressing feather samples, taken from adult shrikes obtained during the breeding season across our study range, against δ^2^H_p_. As all shrikes molt their first primary flight feathers on or near their breeding sites prior to fall migration [13,14], and maintain flight feathers until after subsequent reproduction the next year, the δ^2^H_f_ values of these feathers represented our best sample of potential known-origin samples.

We assessed whether age contributed to variation in shrike δ^2^H_f_ values using a General Linear Model with δ^2^H_p_ as the predictor variable and δ^2^H_f_ and age as factors, allowing interactions among variables. Based on AICc model selection, the most parsimonious model was δ^2^H_p_ = δ^2^H_f_ + age [14]. Therefore, we created separate isotopic surfaces for each age class (ASY vs. SY) independently.

Dispersal among bird populations may occur even over long distances [29-31], potentially negating our assumption that all breeding birds were sampled from their true population of origin. Therefore, we also attempted to identify and remove outliers that were likely dispersers prior to creating our δ^2^H_f_ isoscapes. Thus, we ran a preliminary analysis and removed outliers that had residuals greater than three standard deviations from the mean. After removing outliers (n = 10), the derived regression equations describing the relationship between δ^2^H_f_ and δ^2^H_p_ for shrikes were as follows: SY: δ^2^H_f_ = -1.27 + 1.04* δ^2^H_p_; ASY: δ^2^H_f_ = 2.03 + 1.04* δ^2^H_p_. These regression equations were used to derive final feather isoscapes for SY and ASY shrikes by recalibrating the δ^2^H_p_ isoscape using the raster calculator in Spatial Analyst^TM^ (ESRI, Redlands, CA). We then interpolated the residuals from these regression equations using a 3^rd^ order global polynomial giving a smooth trend surface (RMSE = 11.06) and added to this layer to adjust for regional variation in the relationship between δ^2^H_f_ and δ^2^H_p_ [26,28]. The final δ^2^H_f_ isoscapes were delimited by the known current breeding range of the species by masking the surface with an overlay of the species’ range (http://www.cec.org/), with manual adjustments in the northeastern portion of the range based on data from the Ontario Breeding Bird Atlas [32].

*Geographic Assignments to Origins*

We assigned our validation sample set of 32 SY and 70 ASY individuals to a breeding-origin individually by assessing the likelihood that cells within the appropriate (SY or ASY) δ^2^H_f_ isoscape could have represented a potential origin for a sample given the measured δ^2^H_f_. Specifically, we used a normal probability density function [19,33] to estimate the likelihood that any given cell within the isoscape represented a potential origin for the sample. In brief, this method compares the observed δ^2^H_f_ against a mean expectation (cell value) given error, which we estimated based on the standard deviation of the residuals of our best general linear model (σ =10.05‰). For assignments to origin conducted based on δ^2^H_f_ alone, the resulting probability densities were normalized by dividing by the sum of the densities across the surface to yield the probability of origin map. We also applied genetic admixture coefficients as priors using Bayes’ theorem [19,33] to estimate posterior probability densities to derive probability of origin maps conditional on probability of belonging to a given genetic subpopulation.

Probability of origin maps for each sample were reclassified to likely versus unlikely origins for a sample by determining the odds that any given assigned geographic origin was correct relative to the odds that it was incorrect. Based on 2:1 odds that a given bird had truly originated from within the range defined by the odds ratio, we identified the set of raster cells that defined the upper 67% of estimated “probabilities of origin” and coded those as 1, and all others as 0, resulting in one binary map per individual. We also conducted analyses using 3:1 odds, which identified the set of raster cells that defined the upper 75% of estimated probabilities of origin. A bird was classified as being ‘correctly’ assigned if the coordinates of its capture location fell within the range defined by the odds ratio (i.e. the geographic area represented by raster cells coded as 1). All regression analyses were done in JMP 8 [34]. Assignment of wintering origin birds using deuterium feather values was undertaken using functions within the R statistical computing environment using the ‘raster’ package [35].

We used Pearson’s c^2^ tests in JMP 8 [34] to assess the null hypothesis that the observed number of correctly assigning individuals, as defined above, did not vary significantly from that expected (i.e. 67% on average using 2:1 odds and 75% on average using 3:1 odds). We also compared observed versus expected rates of correct assignment using a 100 km, 200 km, 400 km and 2000 km ‘buffer’ zone around the probable area of origin identified by our models.

**Results**

Genetic data were obtained from 767 breeding shrikes (Table S1). Results from STRUCTURE [4] suggested that the model *k* = 5 best described the genetic structure of samples within our study area, with the five genetic groups spatially structured in North America [14]. In total, 653 (85.14%) of 767 breeding-season origin samples assigned with high probability (>80%) to a genetic cluster. Using the samples that assigned with high probability to a genetic cluster as ‘training samples’, 71 (70%) of the individuals in our validation sample set assigned to the genetic cluster coinciding with their area of capture (Table S2). Eighteen (≈ 18%) of the individuals not assigned to an origin based on their genetic admixture coefficients were sampled in suspected hybrid zones between genetic clusters (i.e. Arkansas, Mississippi and North Dakota sample locales) and another 6 (≈ 6%) were sampled on the edge of a putative contact zone (i.e. Florida, Tennessee and Southern Texas sample locales) (Table S2).

Feather δ^2^H values derived from breeding SY and ASY shrikes revealed a continent-wide latitudinal pattern of variation in δ^2^H_f_ with little longitudinal resolution [14]. There was little difference in the number of either SY or ASY birds assigning ‘correctly’ using the 2:1 versus 3:1 odds ratio, with slightly more individuals assigned correctly to an area of origin based on 3:1 odds (Table S2), which can be attributed to the larger geographic area representing the individual’s probable area of origin that results from using the 3:1 odds (Table S3). In both models, there was no significant difference between the observed and the expected number of individuals assigned to within the area circumscribed by the probability density model (67% for 2:1 odds and 75% for 3:1 odds) (Pearson’s c^2^, p<0.05) (Table S4). Using 2:1 odds and a 100 km or greater buffer, significantly more ASY birds assign than expected (Pearson’s c^2^, p<0.05) (Table S4). Using 3:1 odds, significantly more ASY birds were assigned ‘correctly’ than expected within a 400 km or larger buffer (Table S4). Within the area encompassed by a 200 km buffer zone around the probable area of origin, 84% of SY assigned correctly using 2:1odds and 94% using 3:1 odds (Table S4). Eighty-three and 87% of ASY birds assigned correctly within a 200 km buffer zone using 2:1 and 3:1 odds, respectively (Table S4). When the buffer zone is increased to 400 km, the capture coordinates of 87% and 94% of ASY birds in the validation sample set assigned to their expected area of origin, using 2:1 and 3:1 odds, respectively (Table S4).

**Supplementary References**

1. Coxon A, Chabot AA, Lougheed SC, Dávila JA, White BW (2011) Characterization of 17 microsatellite loci from the Loggerhead Shrike (*Lanius* *ludovicianus*). Conserv Genet Resour. Online First. DOI 10.1007/s12686-011-9585-2.

2. Mundy NI, Winchell CS, Burr T, Woodruff DS (1997) Microsatellite variation and microevolution in the critically endangered San Clemente Island Loggerhead Shrike (*Lanius ludovicianus mearnsi).* Proc R Soc Lond Series B 264: 869-875.

3. Hansson B, Bensch S, Hasselquist D, Lillandt BG, Wennerberg L, et al. (2000) Increase of genetic variation over time in a recently founded population of great reed warblers (*Acrocephalus arundinaceus*) revealed by microsatellites and DNA fingerprinting. Mol Ecol 9: 1529-1538.

4. Pritchard JK, Stephens M, Donnelly P (2000) Inference of population structure using multilocus genotype data. Genetics 155: 945–959.

5. Chen C, Durand E, Forbes E, François O (2007) Bayesian clustering algorithms ascertaining spatial population structure: a new computer program and a comparison study. Mol Ecol Notes 7: 747-756.

6. Rosenberg NA, Pritchard JK, Weber JL, Cann HM, Kidd KK, et al. (2002) Genetic structure of human populations. Science 298: 2381-2385.

7. Durand E, Jay F, Gaggiotti OE, François O (2009) Spatial inference of admixture proportions and secondary contact zones. Mol Biol Evol 26: 1963-1973.

8. Evanno G, Regnaut S, Goudet J (2005) Detecting the number of clusters of individuals using the software STRUCTURE: a simulation study. Mol Ecol 14: 2611-2620.

9. Jakobsson M, Rosenberg NA (2007) *CLUMPP*: a cluster matching and permutation program for dealing with label switching and multimodality in analysis of population structure. Bioinforma 23: 1801-1806.

10. Coulon A, Fitzpatrick JW, Bowman R, Stith BM, Makarewich CA, Stenzler LM, Lovette IJ (2008) Congruent population structure inferred from dispersal behaviour and intensive genetic surveys of the threatened Florida scrub-jay (*Aphelocoma* *coerulescens*). Mol Ecol 17: 1685-1701.

11. Fedy BC, Martin K, Ritland C, Young J (2008) Genetic and ecological data provide incongruent interpretations of population structure and dispersal in naturally subdivided populations of white-tailed ptarmigan (*Lagopus leucura).* Mol Ecol 17: 1905-1917.

12. Row JR, Blouin-Demers G, Lougheed SC (2010) Habitat distribution influences dispersal and fine‐scale genetic population structure of eastern foxsnakes (*Mintonius gloydi*) across a fragmented landscape. Mol Ecol 19: 5157-5171.

13. Pérez G, Hobson KA (2006) Isotopic evaluation of interrupted molt of northern breeding populations of the loggerhead shrike. Condor 108: 877-886.

14. Chabot AA (2011) The impact of migration on the evolutionary and conservation of an endemic North America passerine: loggerhead shrike (*Lanius ludovicianus*). Kingston: Dissertation, Queen’s University.

15. Wassenaar LI, Hobson KA (2003) Comparative equilibration and online technique for determination of non-exchangeable hydrogen of keratins for use in animal migration studies. Isot Envir Health Stud 39: 1-7.

16. Wassenaar LI (2008) An introduction to light stable isotopes for use in terrestrial animal migration studies. In: Tracking animal movements with stable isotopes. Hobson KA, Wassenaar LI, editors. New York: Academic Press. pp. 21-44.

17. Bowen GJ, Wassenaar LI, Hobson KA (2005) Global applications of stable hydrogen and oxygen isotopes to wildlife forensics. Oecologia 143: 337-348.

18. Wassenaar LI, Hobson KA (2006) Stable hydrogen isotope heterogeneity in keratinous materials: mass spectrometry and migratory wildlife tissue sampling sampling strategies. Rapid Commun Mass Spectrom 20: 1-6.

19. Royle JA, Rubenstein DR (2004) The role of species abundance in determining breeding origins of migratory birds with stable isotopes. Ecol Appl 14: 1780-1788.

20. Kelly JF, Ruegg KC, Smith TB (2005) Combining isotopic and genetic markers to identify breeding origins of migrant birds. Ecol Appl 15: 1487-1494.

21. Norris DR, Marra PP, Bowen GJ, Ratcliffe LM, Royle JA, et al. (2006) Migratory connectivity of a widely-distributed songbird, the American redstart (*Setophaga* *ruticilla*). Ornithol Monogr 61: 14-28.

22. Boulet M, Norris DR (2006) The past and present of migratory connectivity. Ornithol Monogr 61: 1-13.

23. Hobson KA (2008) Applying isotopic methods to tracking animal movements. In: Tracking animal migration using stable isotopes. Hobson KA, Wassenaar LI, editors. London: Academic Press. 45-78 pp.

24. Langin, KM, Reudink MW, Marra PP, Norris DR, Keyser K, et al. (2007) Hydrogen isotopic variation in migratory bird tissues of known origin: implications for geographic assignment. Oecologia 152: 449-457.

25. Wunder WB (2009) Using isoscapes to model probability surfaces for determining geographic origins. In: West JB, Bowen GJ, Dawson TE, Tu KP, editors. Understanding movement, pattern, and process on Earth through isotope mapping. New York: Springer.

26. Lott CA, Smith JP (2006) A GIS approach to estimating the origins of migratory raptors in North America using hydrogen stable isotope ratios in feathers. Auk 123: 822-835.

27. Hobson KA, Wassenaar LI, Bayne E (2004) Using isotopic variance to detect long distance dispersal and philopatry in birds: an example with ovenbirds and American redstarts. Condor 106: 732-743.

28. Bowen GJ, Wilkinson B (2002) Spatial distribution of δ^18^O in meteoric precipitation. Geol 30: 315-348.

29. Koenig WD, Van Vuren D, Hooge PN (1996) Detectability, philopatry and the distribution of dispersal distances in vertebrates. Trends Ecol Evol 11: 514-517.

30. Sutherland GD, Harestad AS, Price K, Lertzman KP (2000) Scaling of natal dispersal distances in terrestrial birds and mammals. Cons Ecol 4: 16.

31. Winkler DW, Wrege PH, Allen PE, Kast TL, Senesac P, et al. (2005) The natal dispersal of tree swallows in a continuous mainland environment. J Anim Ecol 74:1080-1090.

32. Cadman MD, Sutherland DA, Beck GG, Lepage D, Couturier AR, editors (2007) Atlas of the breeding birds of Ontario, 2001-2005*.* Toronto: Bird Studies Canada, Environment Canada, Ontario Field Ornithologists, Ontario Ministry of Natural Resources and Ontario Nature. 706 p.

33. Van Wilgenburg, SL, Hobson KA (2011) Combining stable-isotope (δD) and band recovery data to improve probabilistic assignment of migratory birds to origin. Ecol Appl 21: 1340-1351.

34. SAS Institute Inc. (2008) JMP® 8*.* Cary: North Carolina.

35. R Development Core Team (2008) R: a language and environment for statistical computing. Available: <http://www.R-project.org/>.
